# The effect of insomnia on development of Alzheimer’s disease

**DOI:** 10.1186/s12974-020-01960-9

**Published:** 2020-10-06

**Authors:** Shaghayegh Sadeghmousavi, Mahsa Eskian, Farzaneh Rahmani, Nima Rezaei

**Affiliations:** 1 Neuroimaging Network (NIN), Universal Scientific Education and Research Network (USERN), Tehran, Iran; 2 School of Medicine, Shahid Beheshti University of Medical Sciences, Tehran, Iran; 3 Research Center for Immunodeficiencies, Pediatrics Center of Excellence, Children’s Medical Center, Tehran University of Medical Sciences, Tehran, Iran; 4 Network of Immunity in Infection, Malignancy and Autoimmunity (NIIMA), Universal Scientific Education and Research Network (USERN), Tehran, Iran; 5 Department of Radiology, Washington University in St. Louis, St. Louis, MO USA; 6 Department of Immunology, School of Medicine, Tehran University of Medical Sciences, Tehran, Iran

**Keywords:** Alzheimer’s disease, Sleep, Sleep deprivation, Insomnia, Inflammatory processes

## Abstract

Alzheimer’s disease (AD) is the most common type of dementia and a neurodegenerative disorder characterized by memory deficits especially forgetting recent information, recall ability impairment, and loss of time tracking, problem-solving, language, and recognition difficulties. AD is also a globally important health issue but despite all scientific efforts, the treatment of AD is still a challenge. Sleep has important roles in learning and memory consolidation. Studies have shown that sleep deprivation (SD) and insomnia are associated with the pathogenesis of Alzheimer’s disease and may have an impact on the symptoms and development. Thus, sleep disorders have decisive effects on AD; this association deserves more attention in research, diagnostics, and treatment, and knowing this relation also can help to prevent AD through screening and proper management of sleep disorders. This study aimed to show the potential role of SD and insomnia in the pathogenesis and progression of AD.

## Introduction

Dementia is one of the major causes of disability and mortality and a common disease in the elderly [1]. It is characterized by difficulties with memory, language, problem-solving, and a decline in cognitive level, which affects daily routine and social activities [2]. Dementia has different types including Alzheimer’s disease, vascular dementia, dementia with Lewy bodies (DLB), mixed dementia, frontotemporal lobar degeneration (FTLD), and Parkinson’s disease (PD) dementia [3].

Alzheimer’s disease is the most common type of dementia [3, 4]. Based on the World Health Organization’s (WHO) reports, nearly 50 million people have dementia worldwide. There are about 10 million new cases annually and Alzheimer’s disease (AD) may contribute to 60–70% of the cases [5].

AD is a progressive neurodegenerative disorder characterized by cognitive and non-cognitive disabilities [6]. Symptoms vary among people with AD based on the degree of damage to neurons in different parts of the brain. The common symptoms of AD are memory deficits especially forgetting recent information, recall ability impairment, loss of time tracking, and problem-solving, language, and recognition difficulties [3, 7–9]. Accumulation of extracellular amyloid-beta (Aβ) plaques is a considerable pathological event in AD, along with elevated intraneuronal tau expression and age-related deposition of intracellular tau as neurofibrillary tangles which occurs concomitantly with Aβ deposition in a mutually exacerbating manner [10–12].

Neuroinflammation is also a major element in AD pathogenesis, initiated by and precipitating both Aβ and tau deposition [13–15]. The neuroinflammatory response in AD entails a complex response from recruitment of peripheral immune cells including leukocytes and T cells, glial cell activation, induction of intracellular signaling pathways, and release of inflammatory mediators including interleukin-1 (IL-1), interleukin-6 (IL-6), interleukin-18 (IL-18), tumor necrosis factor-α (TNF-α), interferons (IFN) and interleukin-12 (IL-12). These cytokines are upregulated in signature AD regions and result in neuronal dysfunction or death [16–19].

Normal sleep has been reported to contribute to tissue repair, thermoregulation, homeostatic restoration, memory consolidation processes, and preservation of neuroimmune-endocrine integrity [20, 21].

During sleep, the brain switches periodically between different activity states which are non-rapid eye movement (NREM) sleep and rapid eye movement (REM) sleep [22]. REM sleep is considered important for learning, memory consolidation, neurogenesis, and regulation of the blood-brain barrier function [23–25], while non-REM sleep has been related to the regulation of hormonal release, the lowering of the thermal set point, and is characterized by a reduction of cardiovascular parameters such as blood pressure [20, 26].

Sleep disorders can cause stress, somatic and psychosocial issues, including anxiety, depression, memory problems, chronic diseases such as cardiovascular disease, hypertension, diabetes and cancer, reduced quality of life, and increased mortality. Also, their other impacts are difficulties with work, increased accidents, and having economic burden [27–29]. Sleep disorders are prevailing due to changes in western lifestyle [30] so that their prevalence in the USA was announced to be about 70 million (based on the Institute of Medicine, Committee on Sleep Medicine & Research 2006) [31]. Among sleep disorders, insomnia has the most prevalence in adults. The estimated prevalence of difficulty in initiating and maintaining sleep is about 30% [32].

Insomnia is a sleep disorder in which patients have dissatisfaction with sleep quality or duration, difficulty in falling asleep at night, or waking up too early in the morning and it can lead to daytime fatigue, low energy, difficulty in maintaining attention, and formation of long-term memory [33–35]. Insomnia is frequently associated with neuropsychiatric comorbidities like anxiety, depression, substance use disorder, and comorbidity with other disorders like the presence of pain and psychiatric disorders [36] and also, adults with insomnia are at great risk of hypertension, type 2 diabetes, neurocognitive disorders, depression, and mortality [37–39]. Acute insomnia is defined as any of the mentioned symptoms occurring for less than 4 weeks, which usually resolves with discontinuation of the causal stressor [40]. Untreated acute insomnia or persistence of the stressor can lead to chronic insomnia, which is in association with comorbid anxiety and depression [41].

The human immune system follows diurnal patterns like that of the circadian rhythm [42, 43]. Levels of cytokines and immunoglobulins are highest during the night, while immune cells in the blood are at their highest levels in the early evening, and their lowest blood levels in the morning [44]. Sleep disturbance disrupts this regulation through increasing levels of proinflammatory cytokines such as IL-6, TNF-α, and IL-1, and CRP levels [45, 46]. Indeed, sleep duration is directly correlated with lower levels of inflammatory markers [47–50], and hence with a predisposition to AD.

Because of the fast-growing number of patients with dementia, particularly AD, and the fact that despite all scientific efforts, at the moment treatment is not a feasible option for this disease, since insomnia is a manageable and preventable disorder, understanding the role of insomnia in AD may lead to prevention or treatment opportunities for AD.

Based on the studies, the major pathological agent in AD is Aβ. It has been reported that insomnia can cause a rise in the CSF levels of Aβ [51].

**Fig. 1:**
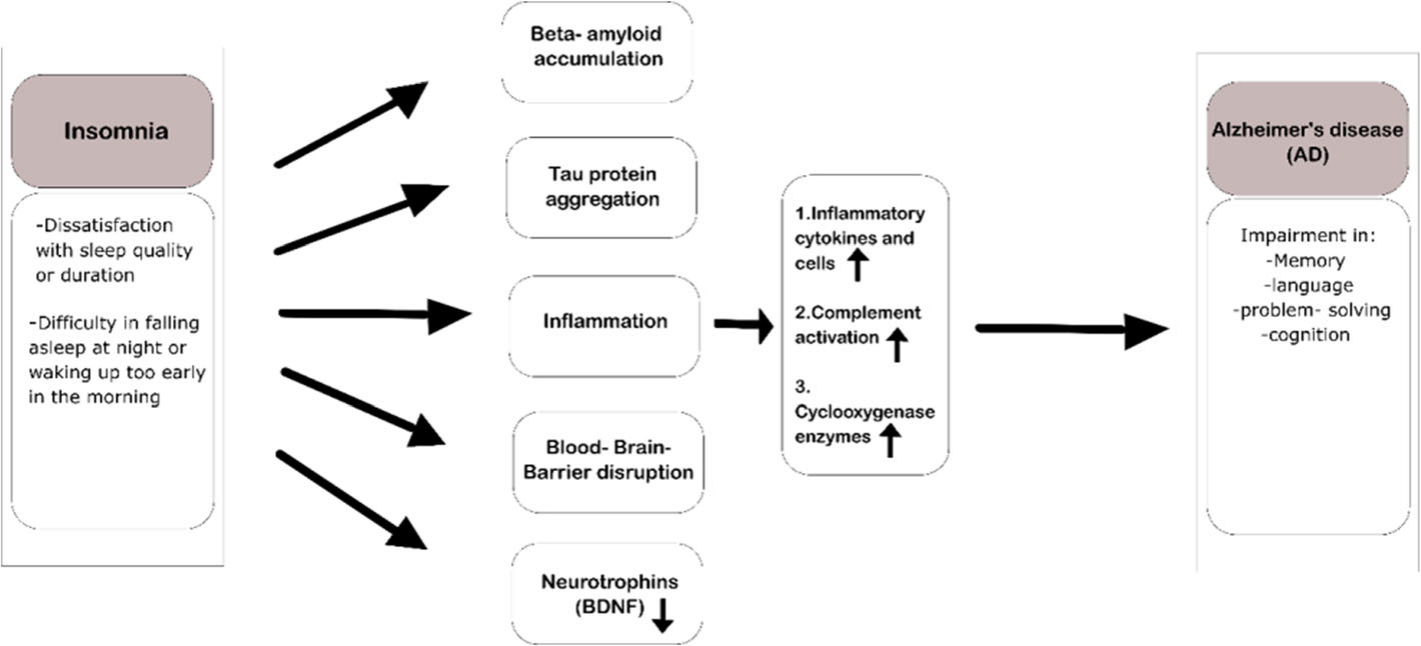
Mechanisms that can link insomnia to the pathogenesis and progression of AD

Sleep deprivation and insomnia can induce aggregation of Aβ peptides and tau proteins, the two hallmark pathological features of Alzheimer’s disease (AD) [52–54]. In light of this and several other findings, we aim to elucidate the potential role of SD and insomnia in the pathogenesis and progression of AD (see Fig. 1).

### Beta-amyloid

Amyloid peptides are 39 to 43 residue proteolytic products [55] which are formed through cleavage of the amyloid precursor protein (APP) by β and γ-secretases. Accumulation of Aβ is a major cause of synaptic dysfunction and impairment of neurotransmission, which is critical to the pathogenesis of AD [56]. Accumulation of Aβ is the result of an imbalance between its production and clearance [57]. Intraneuronal Aβ accumulation is an early event in AD, resulting from cleavage of the APP at the beta cleavage site [58].

β-site APP-cleaving enzyme I (BACE1) is responsible for the Aβ accumulation. APP is a protein located in the plasma membrane that is concentrated at neuronal synapses [57, 59], the Golgi network, endoplasmic reticulum (ER), and endosomal, lysosomal, and mitochondrial membrane [59–61]. It has roles in cell adhesion and movement [62, 63]. Aβ is predominantly produced as a monomer, and then aggregates and forms multimeric complexes [64] at the plasma membrane where β- and γ-secretases are at their highest concentration [65]. The oligomeric species of Aβ are the most pathological components and can cause hippocampal synaptic loss, and failure of long-term potentiation in rats [66–68].

Degeneration of cholinergic neurons, alteration in glutamatergic synaptic transmission, and most importantly synaptic loss, dendritic spine loss, and cell death are among the neurotoxic effects of amyloid-beta peptides [10, 69–72].

Intracerebral injection of synthetic Aβ that included a mixture of amyloid-β fibrils, protofibrils, oligomers, and monomers deteriorated learning behavior in rats [73, 74].

Amyloidosis is a clinical disorder that occurs due to the extracellular and/or intracellular deposition of insoluble pathogenic amyloid built of misfolded proteins [75–78].

Cells have well-designed systems including chaperones to check that protein folding. In addition to this ensuring system, there are selective degradation mechanisms like the proteasome which has a disposing role of the misfolded proteins. Unlike other misfolded proteins, amyloid species can escape from quality control systems such as the ubiquitin-proteasome pathway, due to their ability to aggregate into fibrillar structures [79]. This fact implicates that inhibition of the proteasome pathway, other Aβ degrading enzymes such as β- and γ-secretases [80, 81], or uptaking to lysosomes or brain vasculature [82–85] can lead to the accumulation of misfolded amyloidogenic proteins and peptides. Different cell types in brain parenchyma and vasculature have roles in the cellular clearance of Aβ. Cell surface Aβ-binding receptors mediate these pathways and apolipoprotein E (apoE) regulates them [86, 87].

Several studies demonstrate sleep disturbance and insomnia are associated with a higher incidence of dementia [88–92]. Animal models of AD and sleep disturbance have been demonstrated that Aβ levels in brain interstitial fluid (ISF) and brain tissue remarkably increased in mice suffering from sleep deprivation, compared to normal mice [52, 93–95]. Chen et al. [52], after inducing SD in Sprague-Dawley rats, confirmed that SD impaired cognitive function and increased the levels of brain Aβ peptides, and significantly increased the levels of the BACE1and β-secretase, but had little impact on the levels of Aβ-degradation enzymes. They mentioned that this result may be the main cause of the over-expression of Aβ1-42 and Aβ1-40. Kang et al. [93], by using in vivo microdialysis in mice, found that the amount of ISF Aβ significantly was increased during acute SD, and also chronic sleep restriction (SR) significantly increased Aβ plaque formation in amyloid precursor protein transgenic mice.

Human studies such as Hung et al. reported that patients who have insomnia are at a greater risk of being diagnosed with dementia. During the 3-year follow up of 51,743 primary insomnia patients (older than 20 years of age), after adjusting for sex, the region of residence, and selected comorbidities, a primary insomnia diagnosis was independently associated with a 2.14-fold (*p* value < 0.05) higher risk of subsequent development of dementia [96]. Chen et al. suggested the correlation between Aβ levels of CSF with the duration of insomnia in 23 patients with chronic insomnia aged between 46 and 67 and showed long-term poor sleep quality has accumulative effects on brain Aβ42 levels [51]. It has been suggested that wakefulness increases neuronal activity, and hence the production and secretion of Aβ [97]. Moreover, reduced neuronal activity during sleep can increase the clearance of Aβ and lower Aβ production [93, 98–100]. Also, the glymphatic system, which is a waste clearance system that uses perivascular tunnels made from astroglial cells, to promote efficient removal of soluble proteins and metabolic waste in the CNS, is more active in sleep time compared to wakefulness [101]. A study in this area by Xie et al. revealed that radioactively labeled Aβ injected into the cortex of live mice was removed more efficiently during sleep than in awake time [102].

Based on evidence from animal and human studies, it can be concluded that increasing Amyloid peptides during sleep deprivation may be potential pathogenesis of AD progression. However, more studies are warranted.

### Tau protein

Tau is the major microtubule-associated protein in neurons [103]. Human tau is expressed by the MAPT gene, located on chromosome 17 [104]. In the human CNS, especially in the brain [105], tau protein is translated from an mRNA transcript producing six tau protein isoforms which the amount of them varies in different regions. For example, in humans, the 0N3R tau is lower in the cerebellum in comparison to other brain regions and 4R tau isoforms are increased in the Globus pallidus [106–108]. The morphology and integrity of neurons are maintained largely by the cytoskeleton, which is partially composed of microtubules. The main biological functions of tau are considered to be the stimulation of microtubule assembly and the reduction of their dynamic instability [109–111]. In addition to the mentioned roles, it has been suggested that it may have other physiological functions including interfering with the binding of kinesin and kinesin-like motors to microtubules which cause inhibition of plus-end directed axonal transport in the absence of its phosphorylation by glycogen synthase kinase (GSK)-3 [112]. Also, tau has interactions with mitochondria [113], plasma membrane [114], and nucleic acids [115, 116], showing its act as a mediator between microtubules and these organelles [103]. Tau is a phosphoprotein that has 2–3 moles of phosphates per every mole [117, 118]. The phosphorylation of tau regulates its binding to microtubules and stimulates their assembly. A normal level of phosphorylation of tau is required for optimal function, whereas the hyperphosphorylated tau impairs its biological activity [119–122]. Hyperphosphorylation of amino acids in tau proteins causes the detachment from the microtubules which this dissociation is a prerequisite for them to aggregate, impairment of the axonal transport, starvation of neurons which leads to cell death and synaptic loss [123–126]. Intracellular accumulation of misfolded tau leads to a reduction in the cellular burden of aggregated proteins and also promotes the secretion of tau aggregates [127]. Also, tau secretion is a regulatable process, and dysregulation of it can cause the spread of tau pathology [127–129]. Abnormal hyperphosphorylation of tau and its intracellular aggregation in the brains and extracellular aggregation in CSF and ISF can be detected in AD patients and it is considered as the second pathological hallmark of AD [103, 130–133]. Neurofibrillary tangles (NFTs) are intracellular fibrillar structures composed of aggregations of abnormally phosphorylated tau [134, 135]. The number and localization of NFTs, unlike the senile plaques, are associated with the severity of dementia [136]. As a leading component in AD pathogenesis, hyperphosphorylated tau is considered as a neurotoxic agent that can lead to neuronal loss [137, 138]. Allen et al. [139], studied the neurodegeneration in the transgenic mice expressing isoform of human tau. According to light microscopy, many nerve cells in the brain and spinal cord were strongly immunoreactive for hyperphosphorylated tau and electron microscopy detected the presence of abundant filaments made of hyperphosphorylated tau protein. Dysfunction and death of nerve cells due to mutant tau protein were suggested in their study. In another study, SantaCruz et al. [140] suggested that mice with a repressible human tau variant developed progressive age-related NFTs, neuronal loss, and behavioral impairments. Argyrophilic tangle like inclusions, brain weight loss, neuron loss, brain atrophy, abnormal accumulation of hyperphosphorylated tau lesions, and impairment of spatial memory developed more as the mice aged so that there were no significant abnormalities during the probe trials in the water maze in 1.3-month-old transgenic mice, and the youngest mice showed no major deficits in the retention of spatial memory but the retention of spatial reference memory declined dramatically in an age-dependent manner. After the suppression of transgenic tau, memory function recovered, and neuron numbers stabilized, but NFTs continued to accumulate.

Several mechanisms have been suggested for the role of tau in neurodegeneration including causing the disassembly of microtubules [141], compromising the microtubule stability and function, resulting in a loss or decline in axonal or dendritic transport in disease [142, 143], disrupting intracellular compartments such as mitochondria and the endoplasmic reticulum that are essential for normal metabolism and alteration of the distribution of these organelles due to the change in microtubule-dependent motor proteins [144–146]. It has been suggested that there is an inter-relationship between Aβ and tau and they do not act in isolation [147–149] and either of them has a crucial role in synaptotoxicity and neurodegeneration. Some evidence suggests that tau pathology can be ameliorated to some extent by Aβ immunization which triggers the phosphorylated-tau aggregation in the neuronal processes [150–153]. Also, there is strong evidence that tau is essential for Aβ-mediated pathology in animal models of plaque deposition and genetically decreasing endogenous tau is protective against synapse loss [154–156]. Jackson et al. [157], to examine Aβ-plaque load and synapse loss in the presence of human tau, generated a mouse model of early AD, in which mutant human APP, PS1, and wild-type human tau were co-expressed. They analyzed the interactions of human tau and Aβ in a mammalian brain with age-related pathology and compared it to the group of mice (8–10 months old) with only APP and PS1 gene mutations using western blotting, ELISA, immunohistochemical analysis, and array topography. They found that over-expressing wild-type human tau increases Aβ-plaque size and dystrophic neurite number but it did not cause Aβ-mediated synaptic loss and neuronal loss, while in another study by Umeda et al. [158], wild-type human tau expressing mice with high levels of human Aβ oligomers had neurofibrillary tangle pathology development and synapse loss at much older ages (18 months) which the difference between the result of these two studies can be due to the different ages examined and the role of age in cognitive deficits.

For relating insomnia to the development of AD through tau pathologies, we reviewed the literature addressing this issue.

Likewise, Aβ neuronal activity can induce the extracellular release of tau. Pooler et al. [129] showed that stimulation of neuronal activity results in the release of endogenous tau in an in vitro model by AMPA receptor stimulation. They identified that AMPA receptor stimulation increased tau release in a dose-dependently manner compared with control cells and the amount of extracellular tau was substantially increased. They concluded that this secreted tau might underlie the propagation of tau pathology in tauopathies. Also, Wu et al. [159] found that increased neuronal activity enhances tau pathology in vivo. They optogenetically and chemogenetically stimulated transgenic tau mice and it caused robust worsening of pathology and accumulation of cell body tau in the stimulated hippocampus which appeared to be neurotoxic, and lead to exacerbated hippocampal cell layer atrophy but additional pathology in cells such as the dentate gyrus granule cell layer was not detected. However, in the transgenic mice, tau accumulates slowly in the granule cell layer (more than 18 months to become apparent), so more time may be needed to effectively induce tau propagation into the granule cells of the dentate gyrus and pathology advancement. So based on the aforementioned studies, we can relate increased neuron stimulation caused by sleep deprivation to tau aggregation.

Rothman et al. [94] tested the hypothesis that sleep restriction worsens memory impairments, Aβ, and tau accumulations in the brain in a mouse model of AD. They established sleep restriction (6 h/day for 6 weeks) in transgenic AD mice and compared the analysis data of SR and control groups. Behavioral data indicated deficits in contextual and cued memory in SR mice that were not present in the control group (*p* < 0.04). Both Aβ and tau levels increased in the cortex of SR mice compared to control. Qiu et al. [95] also demonstrated the exacerbation of AD due to the chronic sleep deprivation in AβPP(swe)/PS1(ΔE9) transgenic mice. Mice exposed to 2-month SD in addition to an altered Aβ precursor processing showed an elevated level of phosphorylated tau protein, and impaired cognitive performance as compared to non-sleep deprivation controls and these changes were long-lasting and were irreversible during a 3-month normal housing condition. Di Meco et al. [53] studied the effect of SD on the development of AD in a transgenic mouse model with plaques and tangles (3xTg mice). Compared with controls, the behavioral assessment showed that SD-treated (4 h sleep restrain per day for 8 weeks) mice had a significant decline in their learning and memory and a significant increase in tau protein insoluble fraction which is associated with tau metabolism impairment. Another study by Lucey et al. [160], by using single-channel EEG with PET imaging and CSF analysis of both Aβ and tau in participants enrolled in longitudinal studies of aging, revealed that a decrease in SWS, especially at the lowest frequencies of 1–2 Hz, was more associated with the accumulation of tau even more than that of Aβ. They suggested that changes in NREM SWA might lead to tau pathology and cognitive impairment either before or at the earliest stages of symptomatic AD.

Holth et al. [161] showed that chronic SD increases tau acutely over hours and also drives tau pathology spreading in the brains of mice and humans. In closing, they have mentioned that optimization of the sleep-wake cycle should be considered for the prevention of AD and other tauopathies.

Together, these studies can explain the impact of insomnia on AD development thorough tau pathogenesis and accumulation.

### Inflammatory processes

Inflammation occurs in the AD brain because of the existing damage. Based on the prior section, overproduction of Aβ is the major cause of the AD pathology although Aβ is detected in both normal and AD brains [162] and this suggests that Aβ alone may not be enough to cause AD. Inflammatory proteins have been reported as the potential pathogenesis of AD [163–165]. The AD can predispose the brain toward the occurrence of chronic inflammation to cause more damage besides the primary pathologic events [166]. The inflammatory components that have roles in AD pathogenesis are complement pathway, cytokine and chemokine pathways, cells, cyclooxygenase enzyme, blood coagulation, and fibrinolysis systems, and other acute-phase proteins such as ApoE and free radicals [167]. These different mechanisms lead to a vicious cycle of AD pathogenesis. For example, Aβ accumulations can directly activate the complement proteins [168], then the complement proteins can accelerate the aggregation of Aβ so it is a bidirectional relationship [169, 170] and as more Aβ becomes aggregated better, it can activate complement component (Clq) [171]. Aβ provokes cytokine production [172, 173], in turn cytokine production can lead to stimulation of Aβ precursor protein production [174]. Further Aβ deposition stimulates inflammation consistently.

Chronic inflammation especially increased levels of C-reactive protein and IL-6 have been reported as the potential mechanism of the complications of insomnia [39, 175–177] same as increased incidence of infectious diseases, for instance, pneumonia [178], common cold [179], herpes zoster [180] and HIV [181], inflammatory diseases such as rheumatoid arthritis [182], heart failure [183], cardiovascular disease [184, 185], and cancer [186, 187]. Therefore, insomnia and disturbance of sleep provide a route to the production of inflammatory mediators [45, 188–191]. So, activation of the inflammatory response links insomnia and dementia risk [192, 193]. In human studies, even experimental sleep duration manipulation leads to increases in inflammatory components [175, 194]. Based on Irwin et al.’s studies after a night of partial sleep deprivation, activation of upstream markers of inflammation including activation of inflammatory signaling pathways such as nuclear factor κB (NF-κB), activator protein 1, and STAT family proteins also increase in mRNA expression of other proinflammatory cytokines [48, 176, 194]. Irwin M.R. et al., by assessing the level of intracellular proinflammatory cytokines three times the day and after partial sleep deprivation in 30 healthy adults, showed that in the morning after a night of sleep loss, in addition to a significant increase in monocyte production of IL-6 and TNF-α, more than 3-fold increase in transcription of IL-6 messenger RNA and a 2-fold increase in TNF-α messenger RNA were detected [48]. A meta-analysis study written by Irwin M.R. et al. including 72 studies (total patients number was > 50,000) suggested the association of sleep disturbance and occurring inflammation. This study suggested that sleep disturbance was associated with higher levels of CRP (ES .12; 95% CI = .05–.19) and IL-6 (ES .20; 95% CI = .08–.31) [176].

In the next section, we discuss the common inflammatory components in both AD and insomnia, separately.

#### Inflammatory cytokines and cells

Innate immunity is the first line of defense against tissue damage and microbial infection [195]. Monocytes, macrophages, and dendritic cells are the immune cells of the innate immune system and within minutes to hours after detecting a foreign challenge, become activated, and initiate a cascade of inflammatory processes that activate the key intracellular transcription factors such as NF-κB and activator protein-1 (AP-1). Activation of NF-κB leads to the production of proinflammatory cytokines including TNF and IL-1 that have roles in the inflammatory response [187, 195].

A mechanism that has roles in nocturnal increases in proinflammatory cytokines is an accumulation of hazard signs including reactive oxygen species, nucleotides such as adenosine triphosphate, and heat shock proteins during the wake period which leads to increased production of proinflammatory cytokines and then can support the initiation of adaptive immune responses [196].

Several studies contest the claim that there is neural-immune signaling which suggests the possibility of a homeostatic feedback loop between sleep and inflammation. Dantzer et al., in a review study, showed that the brain can respond to inflammatory stimuli by producing pro-inflammatory cytokines so, during infection or a chronic health problem, the production of the pro-inflammatory cytokines can lead to sickness behavior and depression [197]. In another study, Irwin et al. reviewed the feedback of the innate immune system through the production of cytokines to modulate the brain function [198].

Some studies suggest altered sleep can change the levels of cytokines which are known to be important in regulating inflammation (see Table 1). The first observations which connect sleep deprivation to innate immunity were by Moldofsky et al. and showed prolonged sleep loss or 40 h of wakefulness of 10 healthy subjects led to elevated levels of IL-1-like (*F* = 4.6, df = 2.10, *p* < 0.05) and IL-2-like activity (*F* = 2.2, df = 16.58, *p* < 0.01) [219]. Later studies show that pro-inflammatory biomarker levels including C-reactive protein (CRP), IL-6, IL-1β, IL-17 and IFNγ are elevated during experimental sleep deprivation [177, 220–222]. In a study by Vgontzas et al., it has been suggested that 1 week of sleep deprivation can lead to a significant overall increase in 24-h secretion of IL-6 by 0.8 ± 0.3 pg/ml; *p* < 0.05 and TNF-α by 0.26 ± 0.1 pg/ml; *p <* 0.01. In this study, the impacts of sleep restriction for 7 days in 25 healthy young samples were studied [223]. Another study written by Patel et. al also demonstrated an association between habitual sleep duration and levels of pro-inflammatory cytokines such as CRP, IL-6, and TNFα levels based on the questionnaires completed by 614 individuals [47]. CRP production in the liver can be stimulated by IL-6 so sleep duration has effects on CRP levels secondary [47]. It has been suggested that increased levels of the circulating proinflammatory cytokines may be a result of the activation of monocytic populations which are primary immune sources of IL-6 and TNF [224].

**Table 1:** Association of the pro-inflammatory cytokine with the AD

Cytokine	CNS origin	Aβ deposition	Neurons	Tau protein	Reference
IL-1	Astrocytes, Glial cells, Neurons	↑	Death of neurons ↑ Invasion of neutrophils into CNS ↑ Release of toxins from glial and endothelial cells↑	Tau phosphorylation ↑	[199–203]
IL-6	Microglia, Astrocytes	~	Anti-apoptotic	Tau phosphorylation↑	[204–206]
IL-18	Activated microglia, astrocytes, ependymal cells, neurons	↑	Pro-apoptotic	Phosphorylation of tau↑	[207–209]
IFN-ɣ	T cells, Glia, Neurons	↑	Neural degeneration ↑	phosphorylation of tau↑	[210–213]
TNF-α	Microglia, Astrocytes, Neurons	↑	Pro-apoptotic	Tau phosphorylation↑	[214–216]
IL-12	Glial cells , Activated blood monocytes	Aβ deposition↑	Pro-apoptotic ↑	Tau levels in CSF ↑	[217, 218]

↑: increase

↓: decrease

~: both increase and decrease

Experimental studies describe that elevations in circulating TNFα levels and TNF-α gene expression occur in monocytes following sleep restriction. Irwin et al., by assessing the monocyte proinflammatory cytokine production in 30 healthy adults via DNA microarray analyses during the day and after partial sleep deprivation, suggested the impact of sleep loss on the transcription of proinflammatory cytokine genes and as it is mentioned above sleep loss induced a 2-fold increase in TNF-α messenger RNA [48]. Also, Vgontzas et al. showed that sleep deprivation led to a significant increase in the overall 24-h TNFα secretion (0.26 ± 0.1 pg/ml; *p <* 0.01) in 25 young and healthy sleepers [225]. It has been suggested that in patients with chronic insomnia, chronic alterations in sleep patterns can change the diurnal pattern of cytokine secretion [223, 225, 226]. In a study written by Vgontzas A.N. et al. in 2002 which was discussed above, by assessing 11 insomniacs and 11 healthy controls, it has been shown that IL-6 and TNF are fatigue-inducing cytokines so it is hypothesized that the daytime secretion of IL-6 is negatively influenced by the quantity and quality of the previous night’s sleep [223]. Several studies surprisingly suggest women appear to be especially vulnerable to the effects of sleep loss on cellular inflammation and overproduction of inflammatory biomarkers such as IL-6 and CRP [49, 176, 227].

In sleep disorders and insomnia, blood cell count changes can be seen either. For example, there are studies that demonstrate the decline in natural killer cell (NK) responses. Irwin et al., in a study, demonstrated reduction of NK cell activity to a level 72% of the mean baseline values (p < 0.01) after sleep deprivation in volunteers [228]. Also in another human study, they suggested decreased NK activity, and lower stimulated NK activity, as compared with the controls [229]. In the lymphocyte subsets studies, reduction in cell counts in blood during the night and its decrease during subsequent daytime was shown in sleep, in comparison with continuous wakefulness. This is true for T helper cells, CTL, activated T cells, and monocytes [42, 230, 231].

For more than a decade, studies have indicated that the immune system may have a role in AD; however, inflammation contributes to and exacerbates AD pathology [232–234]. Neuroinflammation that occurs in AD is a complex response that can lead to cellular and molecular changes, recruitment of peripheral immune cells, induction of some intracellular signaling pathways, and release of inflammatory mediators in the brain. All these factors can cause neuronal dysfunction, death, or a combination of both in AD [16, 17].

The hyperexpression of some pro-inflammatory cytokines, including interleukin-1 (IL-1), interleukin-6 (IL-6), interleukin-18 (IL-18), TNF-α, interferon (IFN), and interleukin-12 (IL-12), has been detected in brain and CSF in both animal studies and patients [18, 19].

Based on the aforementioned studies, C-reactive protein (CRP), interleukin-6 (IL-6), IL-1β, IL-17, and TNF are elevated in insomnia and sleep deprivation and there are studies that suggest that sleep and wake changes could increase the risk of cognitive decline [93, 235].

So here are the associations of elevated neuroinflammatory cytokines and chemokine in insomnia with AD pathogenesis:

IL-1 can be produced by glial cells and neurons [6]. In the AD brain, it accumulates in the hippocampus due to stress injury and has a significant effect on hippocampal synaptic function [6, 236]. Based on studies the levels of IL-1, in CSF and/or serum of patients who have AD, are higher than in healthy controls [199, 237–239]. Blum-Degen D. et al. showed that IL-1β was significantly elevated in the CSF of de novo AD patients in comparison to the control group [240]. The early overexpression of IL-1 in AD is related to the proliferation and subsequent loss of dystrophic neuritic elements in Aβ plaques [241], and it clarifies that IL-1 plays a key role in plaque evolution and in particular, IL-1 promotes the synthesis and processing of APP. Hence, it may promote further amyloid production and deposition in plaques which can lead to AD [199, 200, 242–244]. A correlative relationship also seems to exist; the secreted form of APP activates microglia and induces excessive expression of IL-1 [245]. IL-1 activates astrocytes [246] and induces their expression of the secreted acute phase and/or Aβ binding proteins [247]. Therefore, it shows that the production of IL-1 in insomnia and sleep deprivation can make CNS more susceptible to Aβ plaque deposition.

IL-6 principally is produced by activated microglia and astrocytes in different parts of the brain such as frontal, temporal, parietal, and occipital cortex [248, 249]. The high levels of IL-6 can be detected in the CSF of AD patients [240]. As is said before, IL-6 levels are elevated in insomnia and sleep deprivation. Ringheim G.E. et al. showed the enhancement of APP transcription and expression leading to the IL-6/IL-6R-complex in an in-vitro study using human fetal brain tissues were obtained from 14 to 18 week so it indicates a prominent role for IL-6 in Alzheimer disorder [250].

Microglia, astrocytes, and neurons have roles in producing TNF-α [214]. TNF-α can increase the production of amyloid-beta [207, 215] and hyperphosphorylation of tau protein [208].

As stated, BACE1 has roles in the production of Aβ and accumulation of it, and proinflammatory cytokines such as TNFα, IL-1β, and especially IFNγ have been shown that can increase astrocytic BACE1 expression and Aβ secretion in cultured human astrocytes and astrocytic cell lines [251].

Hence, based on what is said, cytokines and cells which are elevated in insomnia and SD can lead to Aβ overproduction and other pathogenesis that occur in AD.

#### Complements

Activation of three complement pathways (classical, lectin, or alternative pathway) has important roles in normal inflammatory responses to injury and can remove the invading microbes, apoptotic cells, tissue debris, and aggregated macromolecules. However, inappropriate complement activation can also lead to injury or death of cells and can be responsible for the disease manifestations [252–254]. Complement has been implicated in different neurological and neuropsychiatric diseases such as depression, epilepsy, demyelination, and dementia which complement leads to inflammation and exacerbates the disease [255–258].

One prominent feature of AD neuropathology is the activation of the classical complement pathway proteins due to the lesions [163]. Aβ and tau-containing neurofibrillary tangles directly activate the full range of classical pathway complement proteins in in vitro studies [168, 259–265]. For example, Litvinchuk A. et al. (2018) suggested that the deletion of C3ar1 in mice leads to a decline in tau pathology, attenuation of neuroinflammation, synaptic deficits, and neurodegeneration [260]. In another study by Boyett K.W. et al., Clq injections into the hippocampus and cortex of transgenic mice lead to increased fibrillar Aβ [266].

Johansson JU et al. assessed the relationship of CR1 genotype, CR1 levels, CR1 structural isoforms, erythrocyte capture of Aβ with AD risk in intravenous blood samples from AD subjects [261] and confirmed that single-nucleotide polymorphisms (SNPs) in the CR1 gene significantly increases the AD risk.

Another study written by Afagh A. et al. showed that C1q is localized almost around plaques containing the β-pleated conformation of amyloid by assessing the tissue distribution and cell association of C1q in the human brain with immunocytochemical double-labeling techniques [259].

The significance of the complement activation in AD has roles in the pathological changes in the terminal stage of AD and the early alternation in the disease course and in vitro studies it has been suggested that Aβ can induce complement-mediated toxicity against neurons in culture [263, 264, 267–270].

The nervous system is susceptible to injury caused by complement dysregulation owing to its poorly protected resident cells including neurons and glia [271, 272]. Potential triggers for this dysregulation of complements and causing neurodegeneration are legion [258].

Studies have shown that the complement pathway is activated during wakefulness [273, 274]. Measuring the immunoglobulins and complement levels in serum can help us to assess the function of the immune system and study the impairment and restorative processes that happen during wakefulness and sleep and the consequences due to sleep loss. A study written by Hui L. et al. suggests that the immunoglobulins and complement components including IgG, IgA, IgM, C3, and C4 of blood samples of healthy volunteers underwent sleep deprivation were increased during 24 h and 48 h sleep deprivation which its mechanism was considered to be through production and release of the cytokines like IL-6. This shows that sleep-wake activity has roles in humeral mediated immunity [275]. Based on the above-mentioned studies, in insomnia and sleep deprivation, increased cytokine production leads to activation of the complement pathway and immunoglobulins secretion thus Aβ increase which is the key role in the pathogenesis of AD.

#### Cyclooxygenase enzyme

Cyclooxygenase enzyme (COXs) are inflammatory agents and enzymes that play roles in the production of the active lipid molecules named eicosanoids, metabolization of the arachidonic acid, and converting them to prostaglandins [276, 277]. In addition to a constitutive isoform (COX-1), which controls the physiological responses and is expressed in the most tissues, a second and inducible isoform (COX-2) is produced in response to injury, growth factors, cytokines, and pro-inflammatory molecules and is responsible for prostanoid production in acute and chronic inflammatory conditions [278, 279].

Overactivation and overexpression of these enzymes have putative roles in AD pathobiology as one of the inflammatory mechanisms [276, 280–282]. In addition to this inflammatory role, it has been shown that they can affect the pathophysiology of the AD via different pathways such as localization of the COX enzyme and PGD2 which is a major metabolic product of COX-2 in the specific cells of the brain including neurons, microglia cells, and astrocytes, specific produced lipids, the interaction of the COXs with intracellular components that are related to AD-like gamma-secretase complex and disruption of hippocampal synaptic function and finally leading to cognitive deficits [276, 283–289]. So it has been demonstrated that COX-2 has roles in the cascade of events that cause neurodegeneration in AD. Indeed, studies show elevation of that COX-2 expression in the AD brain especially in the frontal cortex and the hippocampal formation leads to clinical dementia [281, 290, 291]. In contrast to PGD2, PGE2 has roles in synaptic plasticity, memory, and neuronal protection so it has a protective role [292, 293]. In Alzheimer’s disease, COX-1 is expressed in microglial cells near the Aβ deposits [279] and COX-2 accumulates in neurons [279, 281, 290]. COX-2 expression in neurons can be induced by Aβ, glutamate, and inflammatory cytokines. It has been shown that PGD2 levels are increased in AD [290, 294–296]. It appears that COX-2 expression in the neurons of the hippocampal formation occurs in early AD before even neurodegeneration may happen [281]. Immunocytochemical evidence suggests that the overproduction of COX-2 in the neurons of the hippocampal formation correlates with neuronal atrophy [291]. The COX-2 protein content is increased in neurons with neurofibrillary tangles and in damaged axons [297] and prior to overproduction of the cytokines such as IL-6 and TGF-β1 [298].

Also, in vitro studies show that overproduction of COX-2 in the AD brain may be the result of exposure of the neurons to Aβ, which may contribute to Aβ neurotoxicity [290] so the relation of COX-2 and Aβ may play a key role in mediating the development and progression of AD.

As it is said above, it has been found that prolonged continuous wakefulness and insomnia can lead to impairments in hippocampal long-term synaptic plasticity and hippocampus-dependent memory formation [299]. It appears that SD and insomnia lead to overproduction of COX-2 then increasing in PGD2 and decreasing in PGE2. This suggests that these lipid molecules participate in memory consolidation during REM sleep [300]. For mentioning the effects of these events on neurons and synapse plasticity, a study shows blocking COX-2 by synthetic and soluble Aβ prevents the inhibition of hippocampal long-term plasticity (LTP) and leads to restoration of synaptic function [301].

### Blood-brain barrier disruption

The blood-brain barrier (BBB) is a specialized diffusion barrier and keeps the integrity of the brain by restricting permeability across the brain endothelium layer and has roles in the normal function of the central nervous system [302]. In fact, BBB is a physical barrier via tight junctions between cells, transport barrier via transport mechanisms that can regulate the solute flux, and metabolic barrier with the roles of metabolizing enzymes [302].

The barrier’s function is changeable. It can react to the local changes and requirements and can be modulated by mechanisms and cells in both physiological and pathological conditions [302]. This regulation that occurs in physiological and pathological circumstances can be done by changes in tight junction function [303] and expression and activity of transporters and enzymes [302, 304].

In the normal brain, cerebral endothelial cells control and restrict the entry of leukocytes and circulating agents into the brain. In pathologic conditions, released chemical mediators such as glutamate, aspartate, ATP, endothelin-1, ATP, NO, MIP-2, TNF-a, IL-β1, bradykinin, histamine, thrombin, substance P, platelet-activating factor, and free radicals can increase BBB permeability [305–308]. Some of these molecules are released by endothelium itself and some are released by terminals of neurons that are close to blood vessels such as histamine, substance P, and glutamate, and affect BBB permeability.

A perfect functioning neurovascular unit and BBB are essential for homeostasis and the proper function of neurons [309].

Change in microvascular permeability and the BBB impairment is involved in AD pathology. In several studies, this changed permeability has been detected in the brains of AD patients and it has been considered to be one of the notable events of AD [310–313]. A meta-analysis study by Farrall A.J. based on 31 BBB permeability studies (1953 individuals) of normal aging or with cerebral microvascular disease suggested that AD patients had a greater increase in BBB permeability in comparison to a neurologically healthy control group (26 comparisons, C:S = 510:547, S.M.D. 0.81, 99% CI 0.37, 1.26, *p* < 0.01) [314]. Breakdown in the BBB in AD also has been approved by post-mortem brain tissue studies [315–317]. Bowman G.L. et al. has analyzed the relationships between biochemical markers of BBB integrity, clinical risk factors, CNS IgG synthesis, apolipoprotein E (APOE) genotype, MR-derived white matter hyperintensities (WMH), and volume changes via clinical assessments, brain imaging, CSF and plasma collection, and assessing the CSF–albumin index (CSF-AI) for determining BBB integrity over one year in patients with mild to moderate AD to discover the BBB stability and its functional importance [318]. After a year, BBB disruption was present in an important subgroup of patients with AD (*n* = 8/36, 22%) at all-time points measured.

In AD, the capillary endothelium degeneration, decreased endothelial TJ protein levels, the capillary basement membrane thickening, and degenerating small cerebral arteries can lead to impaired BBB function and cerebral blood flow impairment [319–323]. As it is said above, chemicals such as TNF-a, IL-β1 which can be overexpressed in AD can influence BBB permeability.

In neurologic conditions including multiple sclerosis and AD, the integrity of the BBB can be altered due to the migration of leukocytes into the brain [324, 325]. Leukocyte migration into the brain can cause loss of TJ molecules including occludin and zonula occludens, stimulation of signal transduction cascades, and finally BBB disruption [326].

The BBB disruption and disturbances can lead patients to AD either. Skoog I. et al. investigated BBB function in relation to AD in elderly by the assessment of CSF/serum albumin ratio as a measure of BBB function. They reported that in 85-year-old AD patients, CSF/serum albumin ratios are higher, and the indications of disturbed BBB function were started even before the onset of the disease [327]. The positive relationship between CSF-serum albumin ratio and progression of the disease over 1 year in AD patients also determines that BBB impairment could affect this progress [318]. The dysfunction of the BBB may help substances penetrating through the BBB more rapidly to interact with neurons, and lead to events include involving amyloid accumulation and Alzheimer encephalopathy [323]. So we can understand that the BBB disruption and AD have a bilateral relationship.

Sleep is a homeostatic process. Sleep disruption and insomnia may have negative consequences depending on an acute or chronic course. Many studies of experimental sleep impairments also suggest that sleep loss influences neural functions and cerebral blood flow [328–330]. An animal study written by He J. et al. shows that chronic sleep impairment affects neuroendocrine regulation and changes BBB structure and function directly in mice. They determined how sleep restriction can change the permeability of the BBB based on the brain uptake of sodium fluorescein. After injecting sodium fluorescein, in the control group, BBB had the lowest permeability in the cerebral cortex and it was higher in the brainstem and cerebellum but in the sleep restriction model, the sodium fluorescein uptake was increased in the cerebral cortex [331].

It is shown that as a result of the systemic inflammation and inflammatory mediators overproduction such as TFN-*α*, IL-1β, IL-6, CRP, and COX-2, due to insomnia and sleep loss, we can detect changes in cellular components of the blood-brain barrier, particularly on brain endothelial cells and they can alter the blood-brain barrier permeability [332–337].

So based on this information, we can suggest that sleep deprivation and insomnia can lead to AD due to the BBB disruption.

### The neurotrophins

The neurotrophins (NTs) are essential secreted proteins that bind to specific cell membrane receptors to start signaling pathways and control processes and have multiple functions. They have a widespread expression in the CNS and PNS in both developing and adult brain [338, 339]. Their functions are neuronal differentiation and survival, modulation of neuronal function, axon pathfinding, and synaptic plasticity. NTs can protect the neurons in conditions such as excitotoxic, hypoxic, and hypoglycemic insults [339–347]. NTs have either an instructive or permissive role in the modification of synapses which is activity-dependent. In the instructive role, NTs directly change presynaptic transmitter release, postsynaptic sensitivity, or synaptic morphology, thus this can lead to a persistent synaptic modification. For the permissive role, this modification is developed by other associated factors with neuronal activity, although NTs accomplish functions that are essential for the synapse modification [338].

Nerve growth factors including TGF-β, insulin-like growth factor (IGF), epidermal growth factor (EGF), fibroblast growth factor (FGF), interleukin-6, bone morphogenetic protein (BMP), and platelet-derived growth factor (PDGF) were the first members of the neurotrophin family that were found in the early 1950s as proteins that have roles in sympathetic and sensory neurons survival and growth during development [339, 348, 349]. Among these neurotrophic factors, NT is prominence because of its roles and distribution in the nervous system. In mammals, this family has four structurally related neurotrophins: NGF, brain-derived neurotrophic factor (BDNF), neurotrophin-3 (NT-3), and neurotrophin-4 (NT-4, which also known as NT-4/5) [350]. After the NGF discovery as the prototypic NT, Barde et al. isolated a neuron survival factor from pig brain, which was named brain-derived neurotrophic factor (BDNF) that is homologous in protein sequence to NGF [351]. BDNF is an NT which has a widespread expression in the developing and adult mammalian brain and has been suggested by several studies that have effects on synaptic plasticity and is related to various physiological functions in the brain especially relevant in neuroplasticity, memory and sleep [339, 352, 353]. For example, it was elucidated that BDNF has a crucial role in activity-dependent long-term synaptic plasticity by the impairment of hippocampal long-term potentiation (LTP) in BDNF knock-out mice [354, 355]. Also, Lohof et al. showed that applying exogenously BDNF enhances synaptic efficacy at neuromuscular junctions in culture [356]. Soon thereafter, BDNF, other types of NTs such as NT-3 and NT-4/5 were suggested to promote glutamatergic synaptic transmission in the hippocampus of the mammalian CNS [357–359].

In AD, neurotrophic factors play a protective role in the survival of neurons that are affected by degenerative processes [343, 360]. So changes in the neurotrophic factors and their receptors regulation can cause neurodegeneration and they are observed in both AD animal models and patients [361, 362]. Neurotrophic factors prohibit cell death, cause the neuronal proliferation and maturation, and have roles in the improvement of growth and function of affected neurons in AD [344, 363, 364]. In this context, the neurotrophin BDNF is highlighted.

Reduction by half in BDNF mRNA, its precursor (proBDNF), and mature BDNF concentration have been detected in the entorhinal, frontal, temporal, and parietal cortex, hippocampus, and basal forebrain of AD brains and in mouse models of amyloid pathology [365–373]. Although, because the main source of serum BDNF is cortical, it seems that serum levels of BDNF relatively shows its CNS expression levels [374]. By measuring the serum BDNF, it has been suggested that its reduction happens early in AD progression, prior to plaque deposition in transgenic animals [372] and relates to the degree of cognitive impairment in humans [368]. BDNF is an important regulator of learning and memory processes which is particularly abundant in the prefrontal cortex [342, 375], and reduced BDNF signaling through TrkB leads to impaired spatial memory [376, 377]. Mature BDNF enhances synapses, though proBDNF weakens synapses. This information shows the importance of the ratio of proBDNF to BDNF in synaptic plasticity [378, 379]. Preclinical reports suggest a decrease in cortical BDNF expression in AD models and BDNF-mediated TrkB retrograde transport impairment in neuronal culture can lead to β-amyloid peptides. Also, β-amyloid overproduction can decrease the signaling of BDNF in cortical neurons [373, 380] and can directly inhibit the proteolytic conversion of BDNF from pro-BDNF and cause reducing its levels and by disrupting its axonal transport can modify BDNF levels at the synapses [381]. It has been demonstrated that the binding of BNDF to TrkB can control the production of APP in vitro [382].

As is said above, neurodegeneration of the basal forebrain cholinergic which are the main source of acetylcholine to the cortex and hippocampus can result in AD and they become atrophic in the disease [383, 384]. These neurons have roles in higher CNS functions such as learning, memory, and attention [385, 386]. The loss of cognitive function due to AD has been attributed to the degeneration of this transmitter system [387]. As a consequence of a life-long dependence of basal forebrain cholinergic neurons on the retrograde supply of the NTs for their cholinergic phenotype [388], the relation of NTs to AD has been mentioned.

Sleep has an important effect on cognitive functioning such as the consolidation of synaptic plasticity and long-term memory. Another disorder that has been shown its relation to BDNF and other NTs is sleep deprivation and insomnia. The impact of sleep deprivation on cortical and hippocampal BDNF expression and serum BDNF levels has been elucidated in both animal models and patients [389–392]. Serotonin is the main neurotransmitter which is responsible for the regulation of both sleep-wake circadian cycles and mood states controlling [393]. It is shown that BDNF production is stimulated by serotonin and BDNF improves serotonergic signaling [394, 395]. It has been demonstrated that patients with symptoms of insomnia showed notably decreased serum BDNF levels in comparison with healthy controls which this reduction significantly was related to the severity of insomnia [396, 397]. However, in addition to reduced BDNF levels due to insomnia, wakefulness gave during SD, as an acute stressor for the brain, cause an increase in BDNF content [398, 399]. Therefore, chronic stress leads in long-term sleep disturbance and decrease of BDNF levels while acute stress elevates BDNF levels which became clear in attention to that the acute stress induces glutamate transmission increase which is related to corticosterone receptors activation, whereas chronic stress leads to opposite results.

Therefore, sleep deprivation can lead to BDNF reduction and based on the roles of BDNF and its effects on memory and synapses it can be suggested that through this impairment and reduction, SD and insomnia can result in AD and dementia.

## Aging and cognitive function

As it is mentioned, dementia is a common disease among the elderly [1, 400, 401] but just preclinical impairments may be expected decades before certain diagnosis and cognitive decline may not be seen until discussed mechanisms including the accumulation of amyloid and NFT, inflammatory processes, BBB disruption, loss of synapses, and neuron loss have reached a certain threshold [11, 402–406].

Same as AD, insomnia and SD also are more prevalent in older adults [407–411]. It has been suggested that chronic sleep impairment has effects on cognitive function in the elderly [410, 412–414]and it is considered as a risk factor for the initiation and progression of AD in them [88, 415, 416]. Cricco et al. [417], in a longitudinal study of 6444 men and women (age 65 and older) with chronic symptoms of insomnia who were cognitively intact at baseline, showed that men with chronic insomnia had an increased risk of cognitive decline independent of depression than those free of insomnia (49% more likely) and for women with chronic insomnia, an increased risk of cognitive decline was detected, but only in those who had severe depressive symptoms. In another study by Cross et al. [418], after assessing the association between insomnia disorder and cognitive function among middle-aged and older adults (> 45 years old) by self-report questionnaires, it has been suggested that insomnia disorder in older adults is more associated with impaired memory than adults with insomnia symptoms alone or without any sleep complaints. Also, Haimov et al. [419] explored the association between chronic insomnia and changes in cognitive functioning among older adults (64 older adults without insomnia and 35 older adult insomniacs). They found that older adult insomniacs displayed impaired performance especially in memory compared to older adult good sleepers.

Therefore, based on the related mechanisms that can lead insomnia to the development of AD, and with attention to the high prevalence of insomnia and sleep disorders among the elderly age group, it can be assumed that insomnia has possible effects on AD pathogenesis and cognitive deficit in older adults with Alzheimer’s disease risk.

## Discussion

This review highlights mechanisms by which chronic sleep deprivation and insomnia disorder may result in AD risk. AD is a common progressive neurodegenerative disease characterized by the accumulation of β-amyloid (Aβ) peptides and hyperphosphorylated tau proteins and starts by Aβ and tau deposition [6, 10, 11]. Neuroinflammation is another main part of AD pathogenesis which occurs by the leukocytes and T cells, glial cell activation, and inflammatory mediators release such as IL-1, IL-6, IL-12, IL-18, TNF-α, and IFN. These molecules are overproduced during the disease and can cause neuronal dysfunction or death [13, 16–19]. Regarding to possible correlation between insomnia and AD, we reviewed the common mechanisms in both AD and chronic insomnia.

Aβ is considered as the major pathological agent of AD. It is demonstrated that insomnia can lead to an increase in the levels of Aβ of CSF and this Aβ can cause synaptic dysfunction and neurotransmission impair, essential mechanisms to the pathogenesis of AD [51, 56]. Also, sleep impairment can lead to:

1. Induction of the extracellular release of tau [129, 159] which this increase can result in neurodegeneration and neuron loss and can explain the association of insomnia to AD pathogenesis [53, 95, 160, 161].
2. Inflammation through increasing levels of proinflammatory cytokines and inflammatory agents and enzymes such as IL-6, TNF-α, and IL-1, and CRP and COX levels. However, the differences in the characterization of sleep disturbance, different assessment ways used to evaluate sleep disturbance such as sleep quality or other sleep complaints like sleep duration, and various markers of inflammation have led to not establishing confident results about the relation between sleep disturbances and inflammation.
3. The BBB disruption [334, 335] and 4. Declining in the neurotrophins levels which are essential proteins for neuron survival, modulation of neuronal function, and synaptic plasticity and have roles in neuroplasticity, memory, and sleep [339, 352, 353]. Based on mentioned papers, these conditions due to insomnia can provide a basis for being affected by AD.

In addition to declared events that link these two disorders, it is important to mention that both insomnia and AD are more prevalent in the elderly [1, 400, 401, 407, 408]. The pathogenesis of AD begins years earlier before the cognitive decline occurrence [11, 402–406]. So based on the impacts of insomnia on AD development, chronic sleep disorders in the elderly can deteriorate cognitive function in them [410, 412–414], and [417] can exacerbate the symptoms of AD [88, 415, 416].

Based on these similarities between the pathophysiology of insomnia and mechanisms that cause AD including accumulation of Aβ, inflammation, and other components which are discussed in this review, insomnia disorder can be linked to AD risk [420–422] and this could be a novel target for treatments or prevention AD development and/or resolve the cognitive decline in patients with AD in the future [423].

## Conclusion

This study provides information suggesting SD and insomnia possible effect on memory impairments and AD neuropathogenesis and development by exacerbating important biochemical processes. Therefore, the correction of sleep disorders including insomnia and sleep deprivation could be a potential therapeutic strategy for individuals with AD risk.

## Data Availability

Not applicable.

## Abbreviations

AD: Alzheimer’s disease
Ap-1: Activator protein 1
APOE: Apolipoprotein E
APP: Amyloid precursor protein
Aβ: Amyloid-beta
BACE-1: β-site APP cleaving enzyme I
BBB: Blood-brain barrier
BDNF: Brain-derived neurotrophic factor
BMP: Bone morphogenetic protein
CNS: Central nervous system
COX: Cyclooxygenase enzyme
CRP: C-reactive protein
CSF: Cerebrospinal fluid
DLB: Dementia with Lewy bodies
EGF: Epidermal growth factor
ER: Endoplasmic reticulum
FGF: Fibroblast growth factor
FTLD: Frontotemporal lobar degeneration
GSK-3: Glycogen synthase kinase
IFN: Interferons
IGF: Insulin-like growth factor
IL: Interleukin
ISF: Interstitial fluid
LTP: Long-term plasticity
LTP: Long-term potentiation
NF: Neurotrophin
NFT: Neurofibrillary tangle
NF-κB: Nuclear factor-^k^B
NK: Natural killer cell
NREM: Non-rapid eye movement
PD: Parkinson’s disease
PDGF: Platelet-derived growth factor
REM: Rapid eye movement
SD: Sleep deprivation
SR: Sleep restriction
SNP: Single-nucleotide polymorphism
TNF: Tumor necrosis factor
WHO: World Health Organization
WMH: White matter hyperintensities
